# BMP Antagonist Gremlin 2 Regulates Hippocampal Neurogenesis and Is Associated with Seizure Susceptibility and Anxiety

**DOI:** 10.1523/ENEURO.0213-23.2024

**Published:** 2024-10-16

**Authors:** Nicolette B. Frazer, Garrett A. Kaas, Caroline G. Firmin, Eric R. Gamazon, Antonis K. Hatzopoulos

**Affiliations:** ^1^Vanderbilt Brain Institute, Vanderbilt University, Nashville, Tennessee 37232; ^2^Division of Genetic Medicine, Department of Medicine, Vanderbilt University Medical Center, Nashville, Tennessee 37232; ^3^Division of Cardiovascular Medicine, Department of Medicine, Vanderbilt University Medical Center, Nashville, Tennessee 37232

**Keywords:** anxiety, BMP signaling, epilepsy, Gremlin 2, hippocampal neurogenesis

## Abstract

The Bone Morphogenetic Protein (BMP) signaling pathway is vital in neural progenitor cell proliferation, specification, and differentiation. The BMP signaling antagonist Gremlin 2 (Grem2) is the most potent natural inhibitor of BMP expressed in the adult brain; however its function remains unknown. To address this knowledge gap, we have analyzed mice lacking Grem2 via homologous recombination (*Grem2^−/−^*). Histological analysis of brain sections revealed significant scattering of CA3 pyramidal cells within the dentate hilus in the hippocampus of *Grem2^−/−^* mice. Furthermore, the number of proliferating neural stem cells and neuroblasts was significantly decreased in the subgranular zone of *Grem2^−/−^* mice compared with that of wild-type (WT) controls. Due to the role of hippocampal neurogenesis in neurological disorders, we tested mice on a battery of neurobehavioral tests. *Grem2^−/−^* mice exhibited increased anxiety on the elevated zero maze in response to acute and chronic stress. Specifically, male *Grem2^−/−^* mice showed increased anxiogenesis following chronic stress, and this was correlated with higher levels of BMP signaling and decreased proliferation in the dentate gyrus. Additionally, when chemically challenged with kainic acid, *Grem2^−/−^* mice displayed a higher susceptibility to and increased severity of seizures compared with WTs. Together, our data indicate that Grem2 regulates BMP signaling and is vital in maintaining homeostasis in adult hippocampal neurogenesis and structure. Furthermore, the lack of Grem2 contributes to the development and progression of neurogenesis-related disorders such as anxiety and epilepsy.

## Significance Statement

Regulation of adult neurogenesis via Bone Morphogenetic Protein (BMP) signaling is important in various neurological disorders. Gremlin 2 (Grem2) is a secreted protein regulator of BMP signaling with strong inhibitory potential due to its unique formation of daisy-chain polymers with BMP ligands. However, despite being highly expressed in the hippocampus, the role of the BMP inhibitor Grem2 in hippocampal structure and function is unknown. This paper provides the first evidence that Grem2 is necessary for proper BMP signaling and hippocampal morphology and neurogenesis. Furthermore, we found increased stress-induced anxiety and seizure susceptibility phenotypes in mice lacking Grem2. Together, our data introduce a novel molecular mechanism of hippocampal homeostasis and putative therapeutic target of neurological disorders.

## Introduction

Epilepsy and anxiety are two of the most common neurological disorders affecting the human population. Combined, they account for nearly 2% of the disability-adjusted life years as determined by the World Health Organization (C. J. L. Murray et al., 2012). Anxiety comorbidity is high in the epileptic population, affecting ∼20% of all patients—a rate twice that found in nonepileptic subjects (Brandt et al., 2010; Kanner, 2011; Dworetzky, 2017).

The hippocampus plays a central role in both epilepsy and anxiety. For example, seizure activity in many cases of mesial temporal lobe epilepsy (mTLE) originates from hippocampal–entorhinal interactions (Spencer and Spencer, 1994; Engel, 1996; Bertram, 1997; Ang et al., 2006). In addition, ∼80% of patients with mTLE have structural hippocampal abnormalities (de Tisi et al., 2011; Blumcke et al., 2017; J. Y. W. Liu et al., 2020). The ventral hippocampus is also known to modulate emotion through its inhibition of the hypothalamic–pituitary–adrenal axis, thereby playing a significant role in stress and anxiety (Herman et al., 1998; Bannerman et al., 2002; Kjelstrup et al., 2002; Fanselow and Dong, 2010; Kheirbek et al., 2013; Strange et al., 2014; Herman et al., 2016; Kjaerby et al., 2016). Furthermore, hippocampal volume is significantly reduced in patients with anxiety disorders (Gurvits et al., 1996; Gilbertson et al., 2002; Villarreal et al., 2002; Z. Wang et al., 2010).

Recent research has implicated postnatal hippocampal neurogenesis in the development and progression of epilepsy and anxiety. Adult-born granule cell dispersion and mossy fiber sprouting are hallmarks of epileptogenesis and are responsible for recurrent seizures following insult in mice (Parent et al., 1997; Ribak et al., 2000; Walter et al., 2007; Kron et al., 2010; Cho et al., 2015). In mood disorders, chronic stress causes a decrease in hippocampal neurogenesis (Gould et al., 1992, 1997; Czéh et al., 2002, 2007; Malberg and Duman, 2003; Kim et al., 2004; Zeng et al., 2007; F. Murray et al., 2008; Kronenberg et al., 2009; Spritzer et al., 2011), whereas ablation of neurogenesis causes heightened anxiety (Revest et al., 2009; Schloesser et al., 2009; Snyder et al., 2011; Yun et al., 2016). Hippocampal neurogenesis is enhanced by anxiolytic medications in both rodent models and humans and is necessary to alleviate anxiety (Malberg et al., 2000; Duman et al., 2001; Santarelli et al., 2003; Surget et al., 2008, 2011; Boldrini et al., 2009; Perera et al., 2011).

An important regulator of adult neurogenesis is the Bone Morphogenetic Protein (BMP) pathway. BMPs belong to the Transforming Growth Factor-β superfamily of secreted ligands that regulate expression of target genes such as the Id family of transcriptional repressors (Yadin et al., 2016). BMP signaling is key for proper development of the nervous system, as well as regulation of proliferation and differentiation of many cell types throughout the body (Hemmati-Brivanlou and Thomsen, 1995; Kobayashi et al., 2005; Stewart et al., 2010). Aberrant BMP signaling regulation has been associated with many disease phenotypes (S-N. Wang et al., 2001; Feeley et al., 2005; Katsuno et al., 2008; Yang et al., 2008; Lowery and de Caestecker, 2010; Cogan et al., 2012; Ehata et al., 2013; Sanders et al., 2016; van der Bruggen et al., 2016; Wu and Hatzopoulos, 2019; Elmasry et al., 2021).

BMP signaling is regulated in part by a large family of secreted antagonists that belong to the “differential screening-selected gene aberration in neuroblastoma” (DAN) family of ligands that directly bind BMPs, interfering with their ability to interact with BMP receptors (Walsh et al., 2010; Ali and Brazil, 2014). DAN family antagonists are small, single-domain proteins characterized by a core “DAN” domain (Avsian-Kretchmer and Hsueh, 2004; Kattamuri et al., 2012). Members of the DAN family include DAN, Gremlin, Gremlin 2 (Grem2), Cerberus, and Sclerostin, among others. Grem2 employs a unique daisy-chain polymerization with BMP ligands to block canonical, i.e., Smad-regulated, BMP signaling (Kattamuri et al., 2012; Nolan et al., 2013, 2016). Moreover, Grem2 inhibition of Smad signaling is uniquely accompanied by upregulation of c-Jun N-terminal kinase signaling activation (Tanwar et al., 2014; Bylund et al., 2017). Grem2 has been implicated in bone development, tooth agenesis, and obesity, as well as the regulation of inflammatory response following myocardial infarction (Ideno et al., 2009; Vogel et al., 2015; Sanders et al., 2016; Mostowska et al., 2018; W. Liu et al., 2022). However, its role in the brain has not been identified. In this study, we show that Grem2 regulates BMP signaling in the hippocampus and is vital for proper hippocampal structure and neural stem cell (NSC) proliferation. Importantly, lack of Grem2 is correlated with neurogenesis-related neurological disorders, such as anxiety and epilepsy.

## Materials and Methods

### Mice

Experiments were conducted on 12–16-week-old male and female wild-type (WT) and *Grem2^−/−^* mice. *Grem2^−/−^* mice were generated via homologous recombination to replace exon 2 of the *Grem2* gene locus that contains the entire coding sequence as previously described (Sanders et al., 2016). The *Grem2^−/−^* mice and littermate WT controls were kept on a mixed C57BL/6 and 129/Sv background.

All animals were housed under standard light, temperature, and humidity conditions with *ad libitum* access to food and water. Unless stated otherwise, no significant difference was found between male and female mice as determined by genotype/sex interaction on a two-way ANOVA. All animal procedures were performed in accordance with the Vanderbilt University Medical Center Animal Care and Use Committee's regulations and guidelines.

### Histology

The brain tissue was collected from mice perfused transcardially with saline followed by 4% paraformaldehyde (PFA; Boster Bio, AR1068). After perfusion, brains were excised and postfixed for 2–12 h (hours) in 4% PFA before storage in 30% sucrose at 4°C. Serial sections were cut coronally through the cerebrum approximately from the bregma −0.58 to −3.88 mm at a width of 20 µm and directly mounted onto slides. Sections were air-dried overnight (O/N). Tissue sections were washed with 1× phosphate-buffered saline (PBS) and blocked for 30 min at room temperature (RT) in 1% normal goat serum, 1% bovine serum albumin, and 0.1% Triton X-100. Sections were then incubated in primary antibodies for 90 min at RT or O/N at 4°C. Primary antibodies used were: mouse anti-NeuN (Chemicon, MAB377; 1:500), rabbit anti-phospho-histone-H3 (EMD Millipore, 06-570; 1:400), rabbit anti-Doublecortin (Dcx; Cell Signaling Technology, 4604S; 1:500), rat anti-Sox2 (Invitrogen, 14-9811-82; 1:100), and rabbit anti-Prox1 (EMD Millipore, AB5475; 1:500). Sections were washed with 1× PBS and then incubated with secondary antibodies from Invitrogen Alexa Fluor 488 goat anti-mouse IgG (A11001), Alexa Fluor 555 goat anti-rabbit IgG (A21248), Alexa Fluor 555 goat anti-mouse IgG (A28180), Alexa Fluor 488 donkey anti-rabbit IgG (A21206), and Alexa Fluor 488 goat anti-rat IgG (A11006) at 1:400 dilution in the dark for 1 h at RT or O/N at 4°C. Slides were then incubated for 30 min at RT in DAPI (Invitrogen, D1306; 1:5,000) to stain nuclei. Next, slides were mounted with ProLong Gold antifade reagent (Invitrogen, P36930) and coverslipped. Apoptosis was measured using the Click-iT Plus TUNEL Assay Kit for In Situ Apoptosis Detection (Invitrogen, C10618) following the manufacturer's protocol, including 20 min of proteinase K incubation.

Fluorescent images were captured using a Zeiss Axio Imager Z1 and Aperio Versa 200 (Leica) microscopes. Images were analyzed with Image J and QuPath.

Following stress, brains were processed as described above, sliced (40 μm) using a vibratome, and stored in “antifreeze” buffer (30% ethylene glycol, 30% glycerol, 0.2× phosphate buffer) at −20°C until further use. Free-floating sections were sequentially immunostained using the following primary antibodies and conditions: anti-Nestin (Novus Biologicals, NB100-1604; 1:250) O/N at 4°C followed by 1 h incubation at RT with goat anti-chicken Alexa Fluor 488 (Invitrogen, A-11039; 1:250); anti-phospho-Smad1/5/8 (pSmad1/5/8; Sigma-Aldrich, AB3848-I; 1:2,000) O/N at 4°C followed by 1 h incubation at RT with biotinylated horse anti-rabbit IgG (Vector Laboratories, BA-9010; 1:200); and anti-Sox2 (Invitrogen, 14-9811-82; 1:2,000) O/N at 4°C followed by 1 h incubation at RT with preabsorbed biotinylated donkey anti-rat IgG (Abcam, ab102259; 1:200). Both pSmad1/5/8 and Sox2 signals were amplified using the Vectastain Elite ABC HRP Detection Kit (Novus Biologicals, PK-6100-NB) in combination with tetramethylrhodamine (TSA) and coumarin (Akoya Biosciences, NEL703001KT) or TSA (Akoya Biosciences, SAT702001EA), respectively, in accordance with the manufacturer's instructions. Confocal images were taken on a Zeiss 710 microscope at the Vanderbilt Cell Imaging Shared Resource Core. Quantification was carried out blind to animal sex and genotype using Image J.

### RNA extraction, reverse transcription, and quantitative PCR analysis

Hippocampi from mice were extracted and immediately frozen at −80°C. RNA was isolated using the RNeasy Micro kit (Qiagen) with in-column DNase treatment (Qiagen) following the manufacturer's protocol. RNA concentration was quantified using a NanoDrop Spectrophotometer ND-1000 (NanoDrop Technologies). Reverse transcription was conducted by incubating 100 ng of oligo(dt)15 (Promega) with 3 μg RNA for 5 min at 70°C. A 10 mM of dNTPs (GE Healthcare), 200 U/μl of Mo-MLV reverse transcriptase with 5× associated buffer (Promega), 40 U/μl RNasin (Promega), and water were added to the RNA solution and incubated at 40°C for 1 h, followed by a 5 min incubation at 95°C in order to inactivate enzyme activity. A 1:100 of the final cDNA solution or ∼20 ng served as template for quantitative real-time PCR with GoTaq qPCR Master Mix (Promega) using a C1000 Thermal Cycler (Bio-Rad Laboratories). Gapdh primer pair was used as a control among samples. Primer sequences were as follows: Grem2, 5’-CCTGTCATTCACAGAGAGGA, 3’-CATTCGAGCTCTACGATGAC; Grem1, 5’-ACCCCAGTGAACAAGGCTCTC, 3’-GGAACGGTCAGGCTGAGGACC; Noggin, 5’-GAACATCCAGACCCTATCTTTGAC, 3’-ACATCTGTAACTTCCTCCTCAGCTT; Chordin, 5’-CTAGGAAATGGCTCCCTTATCTATC, 3’-TGTAAGTGACAATGTGTATCCAAGG; DAN, 5’-CTCGCACCCCACTTTCTAGG, 3’-CTCCAAGGTCACAATCTCCCA; Sox2, 5’-AAGGGGAGAGATTTTCAAAGAGATA, 3’-TCATAAAAGTTTTCTAGTCGGCATC; Dcx, 5’-ATGCAGTTGTCCCTCCATTC, 3’-ATGCCACCAAGTTGTCATCA; Bmp2, 5’-CTTCTTCTTCAATTTAAGTTCTGTC, 3’-ATGTCCAAAAGTCACTAGCAAT; Bmp4, 5’-ATGATTCCTGGTAACCGAATGCTG, 3’-CTTCGTGATGGAAACTCCTC; Id3, 5’-CGGGATCCTCTACTCTCCAACATGAAGGCGC, 3’-GCTCTAGACAGGACGACCGGGTCAGTGGCAAAAG; Gapdh, 5’-CTCACTCAAGATTGTCAGCAATG, 3’-GAGGGAGATGCTCAGTGTTGG.

### Western blotting

Tissue samples were homogenized in RIPA buffer containing cOmplete, Mini, EDTA-free Protease Inhibitor Cocktail (Roche) and Phosphatase Inhibitor Cocktail 2 (Sigma-Aldrich). Lysates were incubated on ice for 30 min, vortexing every 2–3 min. Lysates were centrifuged at 16,000 × *g* for 10 min at 4°C, and supernatants were transferred to new tubes. Protein concentrations were measured using the BCA assay. Lysates containing 25 µg of protein were separated by SDS-PAGE and transferred onto PVDF membranes using a Bio-Rad Trans-Blot Turbo transfer system. Membranes were blocked for 2 h at RT using 5% nonfat milk in 1× PBS-T (0.2% Tween 20). Membranes were incubated O/N at 4°C with primary antibody in blocking buffer. Primary antibodies used were rabbit anti-pSmad1/5/8 (Millipore Sigma, AB3848-I; 1:1,000), rabbit anti-Smad1/5/8 (Abcam, EPR25803-154; 1:1,000), rabbit anti-pJnk1/2 (Invitrogen, MA5-14943; 1:1,000), mouse anti-Jnk1/2 (Invitrogen, AH01302; 1:1,000), and mouse anti-Gapdh (Invitrogen, MA5-15738; 1:2,500). The following day, membranes were washed three times for 10 min with 1× PBS-T, incubated in secondary antibodies goat anti-rabbit or goat anti-mouse HRP in blocking buffer for 1 h at RT, washed three times in 1× PBS-T for 10 min, and washed two times in 1× PBS. Blots were visualized using chemiluminescence (Pierce). For quantification, phosphorylated proteins were compared with their nonphosphorylated protein levels that were normalized to Gapdh.

### Behavioral experiments

Mice underwent behavioral testing at 3 months of age. All tests were performed in the light phase of the light/dark cycle within the same 10:00–14:00 h period to account for time-of-day effects. All mice were submitted to only one test per day on a fixed schedule with slated rest days. Mice which experienced stress or chemical challenges were not run on other tests. Mice were acclimated to handling for 7 d prior to testing and acclimated to each testing room for at least 30 min before the start of each test. Every test was run by the same experimenter.

### Locomotor activity

Open-field activity was recorded through the breaking of infrared beams using standard locomotor activity chambers (∼30 cm × 30 cm, ENV-510; Med Associates). Total activity traveled during the 60 min trial was recorded, as was time spent within the center versus the edge during each 5 min bin. Each zone comprised ∼50% of the total surface area of the box. Activity chambers were thoroughly cleaned between each animal with a 70% ethanol solution to minimize olfactory signals left by the previous subject.

### Elevated zero maze (EZM)

The apparatus consisted of an elevated (0.61 m above the floor) annular platform (width 5 cm, diameter 50 cm, wall height 15 cm), constructed of opaque, white plastic, with two opposing enclosed quadrants and two opposing open quadrants (Stoelting). Room lighting was measured at 250–265 lux in the open quadrants and 115–125 lux in the closed quadrants. Mice were placed in an open quadrant at the threshold of a closed quadrant. All mice were allowed 5 min of free exploration. Movement in the maze was automatically recorded via video camera from above using AnyMaze (Stoelting). The total distance traveled and time spent in open and closed quadrants were digitally measured, with entrance into a quadrant defined as 80% of the area of the mouse having entered a new quadrant. The percentage of the time spent in closed quadrants and total distance traveled were analyzed. At the end of the test, mice were returned to their home cages, and the maze was wiped clean using 70% ethanol between each mouse to minimize olfactory signals left by the previous subject.

### Restraint stress

Restraint stress was performed by placing mice into 50 ml polypropylene conical tubes (Corning, 431472) drilled with air holes. Empty space was filled with paper towel scraps until the mouse was unable to move. Mice were held in this restraint for a 1 h period. Mice were run on a 9 day schedule with Day 0 consisting of 1 h restraint stress, followed by a 40 min return to home cage and subsequent run on the EZM (as described above). Days 1–7 consisted solely of 1 h of restraint stress. Day 8 consisted only of a run on the EZM. Restraint stress began each day at 10:00 A.M., and room light levels were maintained at 500–550 lux. To account for differences due to memory running on the same apparatus twice, *Grem2^−/−^* and WT controls followed the same schedule without restraint stress.

### Seizure susceptibility

Mice were injected intraperitoneally with 20 mg/kg of kainic acid (KA; Tocris Bioscience, 487-79-6) and then placed in 15.3-cm-diameter and 30-cm-tall clear cylindrical tubes and video recorded for 75 min. Observed behavior was categorized into levels using a modified Racine scale with correlated EEG analysis (Racine, 1972; Van Erum et al., 2019). Any mouse that experienced a tonic–clonic seizure lasting longer than 2 min was killed. Mice were returned to home cages after 75 min, and the mortality rate from 75 to 120 min was quantified. All mice were killed 120 min postinjection. At the end of the test, the cylindrical stand was wiped clean using 70% ethanol between each mouse to minimize olfactory signals left by the previous subject.

### Novelty-induced feeding suppression (NIFS) test

Mice were food-restricted for 18 h prior to the NIFS test. Mice were then placed in an open field (40 cm × 40 cm × 40 cm) made of opaque, white plastic with no roof. Light levels inside the open-field box were 250–265 lux. There was nothing inside the box except a singular food pellet of normal chow placed in the center. Movement in the box was automatically recorded via video camera from above using AnyMaze (Stoelting). Mice were visually observed in the box for up to 60 min, with the test concluding upon first bite of the food pellet. At the end of the test, mice were returned to their home cages with *ad libitum* access to food and water, and the apparatus was wiped clean using 70% ethanol between each mouse to minimize olfactory signals left by the previous subject. In addition, the pellet was swapped out for a new one at the conclusion of each trial, regardless of whether the mouse took a bite.

### Statistical analysis

Statistical analysis was performed using the GraphPad Prism software (Table 1). Data are represented as the mean ± SEM. Student's two-tailed unpaired *t* test was used for comparison between two groups, Pearson's *χ*^2^ test was used to analyze mortality rates, two-way ANOVA was used to view the interaction between sex and genotype, and the Mantel–Cox test was used to compare time progression of seizure activity between two groups. *p < 0.05, **p < 0.01, ***p < 0.001, and ****p < 0.0001 were considered significant. Where applicable, “*n*” refers to the number of mice and “*N*” refers to the number of brain slices.

**Table 1.** Statistical Table

	Data structure	Statistical test	*p* value
A	Normal	Student's *t* test	0.0371*
B	Normal	Student's *t* test	0.0864
C	Normal	Student's *t* test	0.1160
D	Normal	Student's *t* test	0.6587
E	Normal	Student's *t* test	0.1402
F	Normal	Student's *t* test	0.6007
G	Normal	Student's *t* test	0.0014**
H	Normal	Student's *t* test	0.0003***
I	Normal	Student's *t* test	0.0282*
J	Normal	Student's *t* test	<0.0001****
K	Normal	Student's *t* test	0.0011**
L	Normal	Student's *t* test	0.0017**
M	Normal	Two-way ANOVA	0.1297
N	Normal	Two-way ANOVA	0.2463
O	Normal	Student's *t* test	0.1384
P	Normal	Two-way ANOVA	0.9877
Q	Normal	Student's *t* test	0.0646
R	Normal	Student's *t* test	0.0035**
S	Normal	Student's *t* test	0.0496*
T	Normal	Student's *t* test	0.9365
U	Normal	Student's *t* test	0.0087**
V	Normal	Student's *t* test	0.0302*
W	Normal	Two-way ANOVA	0.0407*
X	Normal	Student's *t* test	0.9624
Y	Normal	Student's *t* test	0.0193*
Z	Normal	Student's *t* test	0.0700
aa	Normal	Student's *t* test	0.0509
ab	Normal	Student's *t* test	0.0043**
ac	Normal	Student's *t* test	0.0007***
ad	Normal	Student's *t* test	0.0200*
ae	Normal	Student's *t* test	0.0960*
af	Normal	Student's *t* test	0.0013**
ag	Normal	Student's *t* test	<0.0001****
ah	Normal	Student's *t* test	0.0182*
ai	Normal	Student's *t* test	0.0335*
aj	Normal	Student's *t* test	0.0403*
ak	Normal	Student's *t* test	0.0440*
al	Normal	Student's *t* test	0.0016**
am	Normal	Student's *t* test	0.0384*
an	Normal	Student's *t* test	0.0328*
ao	Normal	Two-way ANOVA	0.0026**
ap	Normal	Student's *t* test	0.0024**
aq	Normal	Mantel–Cox test	0.0187*
ar	Normal	Pearson's *χ*^2^ test	0.0308*

*p < 0.05, **p < 0.01, ***p < 0.001, and ****p < 0.0001.

## Results

### Grem2 regulates BMP signaling in the hippocampal area

The hippocampus is one of the regions of the brain with high levels of BMP receptors and BMP signaling activity (Charytoniuk et al., 2000; Gobeske et al., 2009; Bond et al., 2014; Yousef et al., 2015). To test whether Grem2 affects BMP signaling in the hippocampus, we isolated and analyzed hippocampal protein extracts from WT and *Grem2^−/−^* mice to assess the activation status of key downstream BMP pathway components using Western blotting. Specifically, we measured levels of the downstream canonical BMP signaling component pSmad and the noncanonical component pJnk because previous studies have shown that Grem2 regulated Smad and Jnk phosphorylation during differentiation of pluripotent stem cells (Tanwar et al., 2014; Bylund et al., 2017).

**Figure 1.**
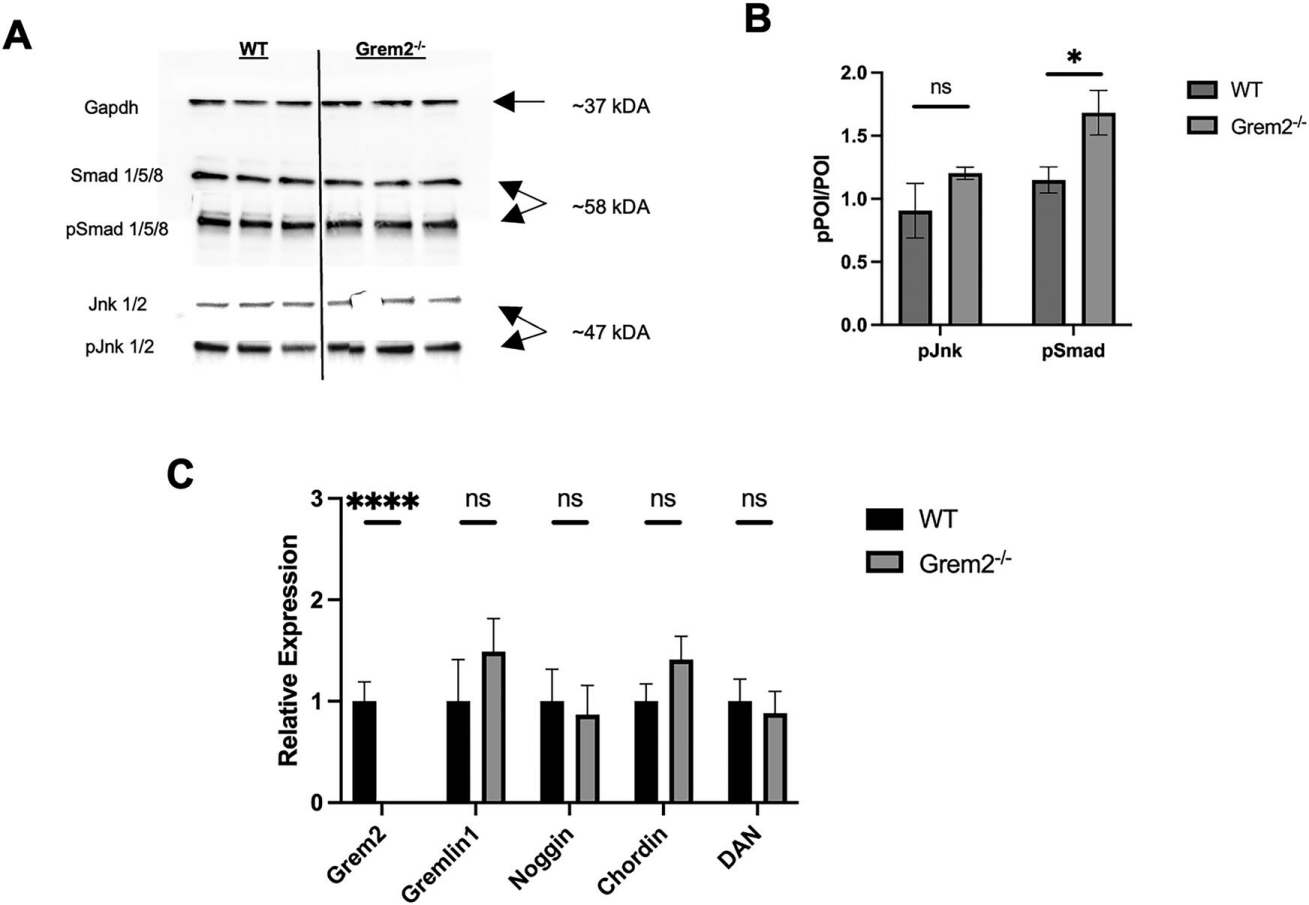
Grem2 regulates BMP signaling in the hippocampus. ***A***, Representative Western blotting images of murine hippocampi protein samples to analyze Smad and Jnk phosphorylation. ***B***, Quantification of blot images shows significantly increase in Smad phosphorylation and modest increase in Jnk phosphorylation in the hippocampi of *Grem2^−/−^* mice. ***C***, PCR analysis of murine hippocampi RNA samples reveals absence of *Grem2* RNA and no compensatory changes in the expression of other DAN family BMP antagonists in *Grem2^−/−^* mice. (p)POI, (phosphorylated) protein of interest.

The Western blotting results showed that hippocampi from *Grem2^−/−^* mice had an increase in Smad1/5/8 phosphorylation (WT, 1.149 ± 0.104 relative expression; *n* = 3; Grem2^−/−^, 1.683 ± 0.177 relative expression; *n* = 3; *p* = 0.0371_a_), suggesting decreased BMP inhibition. In addition, *Grem2^−/−^* mice showed a trend toward an increase in Jnk1/2 phosphorylation (WT, 0.782 ± 0.135 relative expression; *n* = 3; Grem2^−/−^, 0.938 ± 0.140 relative expression, *n* = 3; *p* = 0.0864_b_; Fig. 1*A*,*B*).

qPCR analysis of hippocampal RNA confirmed lack of *Grem2* expression in *Grem2^−/−^* mice (Fig. 1*C*). Moreover, we found that the expression level of *Grem2* in WT mice (27.36 ± 0.70 cycles; *n* = 10) was comparable with that of other antagonists in the hippocampus, including Noggin (Fig. 1*C*). Relative expression levels of other DAN family antagonists (Grem1, 1.488 ± 0.311; *n* = 9; *p* = 0.3737_c_; Noggin, 0.868 ± 0.287; *n* = 9; *p* = 0.7633_d_; Chordin, 1.412 ± 0.231; *n* = 9; *p* = 0.1736_e_; and DAN, 0.880 ± 0.129; *n* = 9; *p* = 0.7042_f_) were not significantly upregulated in the hippocampi of *Grem2^−/−^* mice when compared with WT controls (Fig. 1*C*). These findings suggest that there is no compensation for the lack of Grem2 function by upregulation of other BMP antagonists and indicate that BMP signaling in the hippocampus is altered in the absence of Grem2, underlying the unique role of Grem2 in the adult brain.

### Hippocampal structure is altered in *Grem2^−/−^* mice

**Figure 2.**
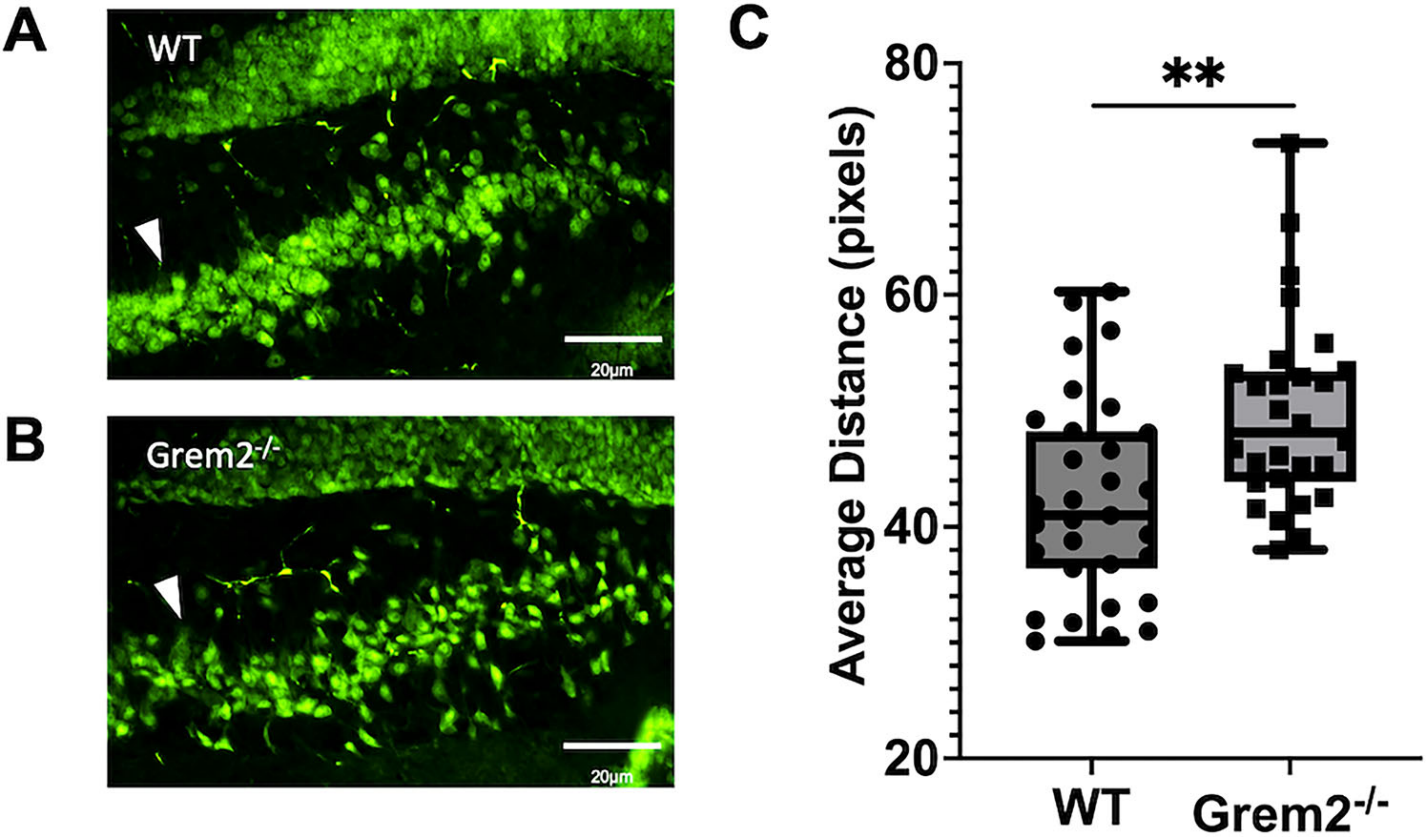
*Grem2^−/−^* mice have structural deficits in the hilar–CA3 region of the hippocampus. ***A***, ***B***, Representative images of the CA3 (arrowhead) as it enters the DG hilus in WT (***A***) and *Grem2^−/−^* (***B***) mice as labeled by NeuN immunofluorescence and examined under 20× magnification. ***C***, *Grem2^−/−^* mice show increased scattering of CA3 pyramidal neurons upon entering the DG hilus (images taken at the bregma −2.055 mm). Extended Data on hippocampal structural deficits are included in Extended Data Figures 2-1–2-3.

To test whether abnormal BMP signaling due to lack of Grem2 is linked to structural deficits in the hippocampus, we stained neurons with NeuN to visualize morphological changes between 3-month-old WT and *Grem2^−/−^* mice. Our results show that the hilar region of the dentate gyrus (DG) exhibited a less structured array of neurons in *Grem2^−/−^* mice compared with WT controls (Fig. 2*A*,*B*). Duplicate staining with Prox1, a granule cell marker, showed that these scattered neurons were pyramidal cells belonging to the CA3 region (Extended Data Fig. 2-1). However, we cannot exclude the possibility that, besides CA3 pyramidal neurons, other NeuN^+^ neurons in the hilus, including interneurons and mossy cells, may also be affected.

10.1523/ENEURO.0213-23.2024.f2-1 Figure 2-1 Prox1 staining shows no evidence of ectopically migrated hilar granule cells in WT or *Grem2^-/-^* mice. [*Images taken at Bregma -1.755  mm*]. Download Figure 2-1, TIF file.

Each NeuN^+^ cell was counted using the cell counter feature on FIJI, and the coordinates of each neuron were plotted on a graph. A linear line of best fit was run through the points, and the perpendicular distance from each point to the line of best fit was calculated utilizing the equation $d_{i}=\frac{\left|y_{i}\;-\;(b\;+\;ax_{i})\right|}{\sqrt{1\;+\;a^{2}}}$ (Extended Data Fig. 2-2). These distances were averaged, and it was revealed that *Grem2^−/−^* mice had a CA3 region with significantly higher scattering within the hilus of the DG compared with WT controls (Fig. 2*C*; WT, 42.38 ± 1.55 pixels; *n* = 9/*N* = 31; Grem2^−/−^, 49.80 ± 1.56 pixels; *n* = 8/*N* = 28; *p *= 0.0014_g_). Gross hippocampal morphology was analyzed by measuring neuronal width at various points along the CA and DG (Extended Data Fig. 2-3), and it was determined that the hilar–CA3 region was the only region displaying significantly abnormal morphology in *Grem2^−/−^* mice (Extended Data Fig. 2-3). Furthermore, comparisons between dorsal and ventral hippocampi revealed increased scatter in both regions, though the effect was more pronounced in dorsal hippocampi (Extended Data Fig. 2-3).

10.1523/ENEURO.0213-23.2024.f2-2 Figure 2-2 **Scatter plots of NeuN^+^ cell location within the dentate hilus and analysis of scatter in dorsal and ventral hippocampi. A-B)** Scatter plots of NeuN^+^ cell location super imposed over flipped hippocampi images from Fig2. Red line denotes example perpendicular distances calculated via equation described in the text. **C)** Increased scatter was seen in *Grem2^-/-^* mice in both dorsal and ventral hippocampi but was only significant in the dorsal hippocampus. Download Figure 2-2, TIF file.

**Figure 3.**
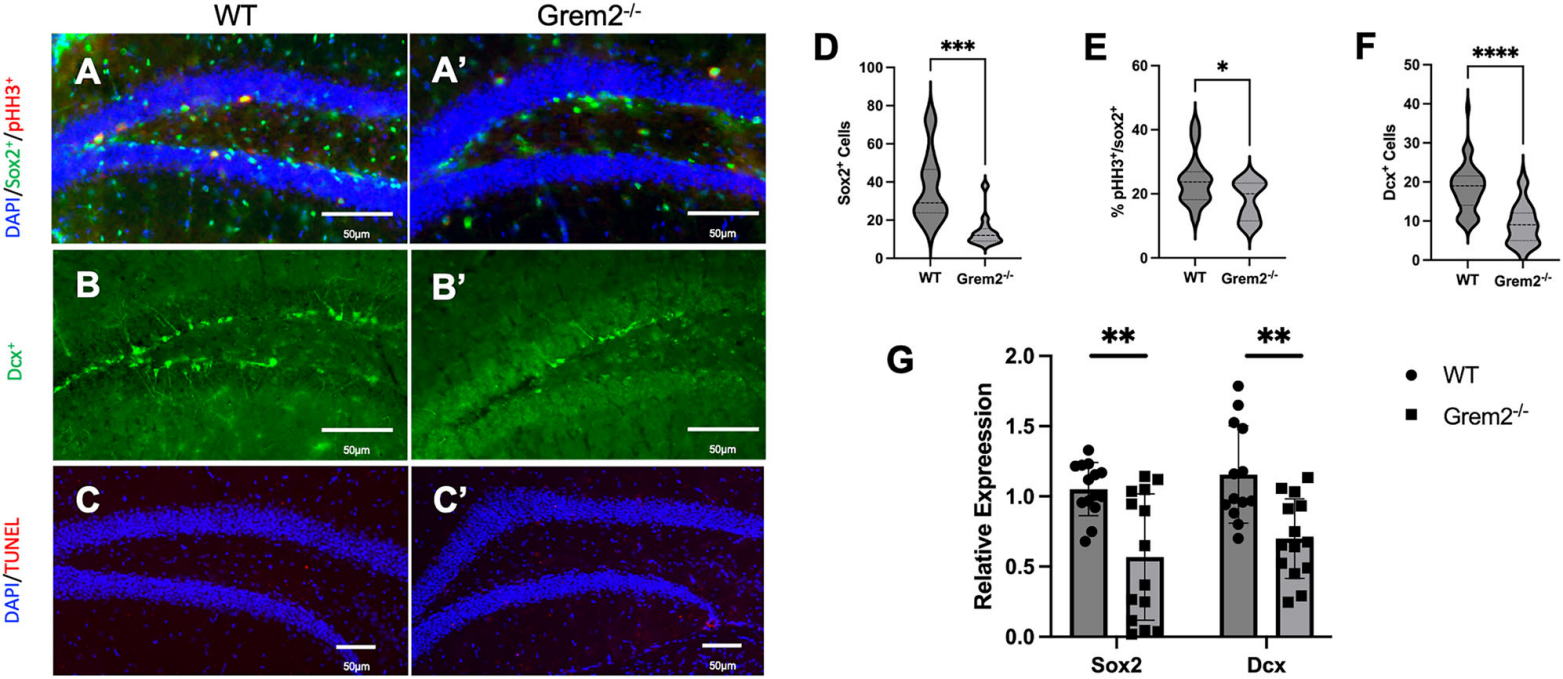
Adult *Grem2^−/−^* mice have proliferative deficits in the SGZ of the DG. ***A–C***, Representative images of murine DG show pHH3^+^, Sox2^+^, Dcx^+^, and TUNEL^+^ immunoreactive cells in WT (***A–C***) and *Grem2^−/−^* (***A’–C’***) mice. ***D***, Quantification of Sox2^+^ cells in the DG of WT and *Grem2^−/−^* mice shows a depletion of Sox2^+^ NSCs. ***E***, Quantification of pHH3^+^ cells indicates that *Grem2^−/−^* mice have a decrease in the percentage of Sox2^+^ NSCs that are actively dividing. ***F***, Quantification of Dcx^+^ cells indicates that *Grem2^−/−^* mice have fewer Dcx^+^ immature neuroblasts in the SGZ. ***G***, qPCR analysis of hippocampal RNA confirmed that there are decreased levels of Sox2 and Dcx gene expression in *Grem2^−/−^* mice compared with WT controls (images taken between the bregma −2.35 and −2.48 mm). Extended Data on ventral versus dorsal hippocampal areas are included in Extended Data Figure 3-1.

10.1523/ENEURO.0213-23.2024.f2-3 Figure 2-3 ***Grem2^-/-^* hippocampal morphology remains intact outside of the hilar CA3 region. A)** Diagram denoting where width measurements were taken along the CA and DG axes. **B**) There is no significant difference in width outside of the CA3c region in *Grem2^-/-^* mice when compared to WT controls. **C-D)** Example images of hippocampi taken at 10x magnification in WT **(C)** and *Grem2^-/-^* **(D)** mice. [*Images taken at Bregma -1.755  mm*]. Download Figure 2-3, TIF file.

### Adult hippocampal neurogenesis is reduced in *Grem2^−/−^* mice

The effect of BMP signaling in neural development has been well recognized, with the release of BMP antagonists from Spemann's organizer in the blastocyst allowing for the specification and development of the entire nervous system (Lamb et al., 1993; Khokha et al., 2005). In addition, BMP signaling has been shown to have a role in adult neurogenesis, with overexpression of BMP ligands causing a decrease in hippocampal adult neurogenesis (Mira et al., 2010; Bond et al., 2012, 2014; Le Dréau, 2022). To test whether the lack of Grem2 affects neuronal stem or progenitor cells, we stained hippocampal tissue sections with an antibody recognizing Sox2, a marker of proliferative NSCs in the subgranular zone (SGZ; Suh et al., 2007), and counted Sox2^+^ cells along the SGZ. Our results revealed that the population of Sox2^+^ NSCs in the DG of *Grem2^−/−^* mice was significantly depleted compared with WTs (Fig. 3*A*,*D*; WT, 37.36 ± 4.93 cells; *n* = 7/*N* = 31; Grem2^−/−^, 14.00 ± 2.26 cells; *n* = 7/*N* = 29; *p* = 0.0003_h_). To determine whether there was also a deficit in the cell cycle progression of these NSCs, we stained hippocampi slices for pHH3, a mitotic marker. We found that the percentage of Sox2^+^ cells that were actively dividing (pHH3^+^ cells) was significantly decreased as well (WT, 24.20% ± 2.07%; *n* = 7/*N* = 31; Grem2^−/−^, 17.94% ± 1.67%; *n* = 7/*N* = 29; *p* = 0.0282_i_), suggesting that not only were there fewer NSCs in the adult hippocampus of *Grem2^−/−^* mice, but additionally, these NSCs were skewed to quiescence compared with WT controls (Fig. 3*A*,*E*). Comparison between dorsal and ventral hippocampal areas showed no significant differences in either WT or *Grem2^−/−^* mice. However, while proliferation was decreased in *Grem2^−/−^* mice in both the dorsal and ventral regions compared with WT mice, this deficit was more prominent in ventral hippocampal regions (Extended Data Fig. 3-1).

10.1523/ENEURO.0213-23.2024.f3-1 Figure 3-1 Proliferative differences are consistent across the dorsal-ventral axis of the DG in *Grem2^-/-^* and WT mice, but ventral proliferative deficits seems to drive overall decrease. A,B) Example images of ventral **(A)** and dorsal **(B)** hippocampi in WT and *Grem2^-/-^* mice stained for DAPI (blue), Sox2 (red), and pHH3 (green). **C)** There is no difference in dividing cells between the dorsal and ventral SGZ in either WT or *Grem2^-/-^* mice. However, the strongest difference appears to be when comparing proliferation within the ventral SGZs. [*Ventral images taken between Bregma -3.28 – -3.48; Dorsal images taken between Bregma -2.055  mm – -2.255*]. Download Figure 3-1, TIF file.

To test whether proliferation deficits in NSCs lead to decreased numbers of immature neuroblasts in the SGZ, we stained the tissues for Dcx, which is a specific marker of these cells. There were significantly fewer Dcx^+^ cells in the DG of *Grem2^−/−^* mice, suggesting that decrease in proliferating NSCs led to a reduction in the number of immature neuroblasts as well (Fig. 3*B*,*F*; WT, 18.28 ± 0.82 cells; *n* = 9/*N* = 89; Grem2^−/−^, 9.11 ± 0.52; *n* = 9/*N* = 100; *p* < 0.0001_j_). In addition, qPCR analysis confirmed that there were decreased levels of *Dcx* gene expression (WT, 1.155 ± 0.096 relative expression; *n* = 13; Grem2^−/−^, 0.699 ± 0.076 relative expression; *n* = 14; *p* = 0.0011_k_) and *Sox2* (*WT*, 1.052 ± 0.050 relative expression; *n* = 14; Grem2^−/−^, 0.567 ± 0.120 relative expression; *n* = 14; *p* = 0.0017_l_) in the hippocampi of *Grem2^−/−^* mice, corroborating the histological findings (Fig. 3*G*).

Though the interaction of sex in the expression of *Sox2* and *Dcx* was not significant (*p*_Sox2 _= 0.1297_m_; *p*_Dcx _= 0.2463_n_), it is notable that male *Grem2^−/−^* mice exhibited more pronounced decreases in Sox2^+^ (average Δsox2^+^ cells = −30.21) and Dcx^+^ (average ΔDcx^+^ cells = −11.04) cells and expression (relative expression_Sox2 _= 0.432, relative expression_Dcx _= 0.582) compared with *Grem2^−/−^* females (average ΔSox2^+^ cells = −16.80; average ΔDcx^+^ cells = −9.81; relative expression_Sox2 _= 0.701; relative expression_Dcx _= 0.714).

Lastly, levels of apoptosis in the hippocampus were analyzed via TUNEL assay. No significant evidence of cell death was detected in the hippocampi of WT and *Grem2^−/−^* mice. There was a low number of TUNEL^+^ cells in the hippocampus of WT or *Grem2^−/−^* mice and no difference between the groups (Fig. 3*C*).

### Grem2 is required for attenuating anxiety in response to stress

Given the decreased proliferation and numbers of neuronal stem and progenitor cells and the abnormal scattering of neurons observed in the hippocampi of *Grem2^−/−^* mice, we tested *Grem2^−/−^* mice for neurobehavioral deficits. Specifically, to evaluate anxiety disorders, mice were run on the EZM platform for 5 min to measure time spent in the closed arms. Although there was a trend toward more anxiogenic behaviors in the *Grem2^−/−^* population, this effect was not significant (WT, 64.38% ± 3.32% time in closed arm; *n* = 28; Grem2^−/−^, 69.13% ± 2.71% time in closed arm; *n* = 26; *p* = 0.1384_o_). Furthermore, there were no sex differences in stress on the baseline EZM (*p* = 0.9877_p_). In addition, *Grem2^−/−^* mice did not display a significant difference in total ambulation in a 1 h open-field locomotor paradigm (Extended Data Fig. 4-1; WT, 2,397.40 ± 235.92; *n* = 25; Grem2^−/−^, 1,835.88 ± 175.44; *n* = 24; *p* = 0.0646_q_). However, the pattern of movement of *Grem2^−/−^* mice was significantly different, with *Grem2^−/−^* displaying more anxiogenic freezing behavior in the first 20 min compared with WT mice (Extended Data Fig. 4-1; WT, 1,655.73 ± 109.70; *n* = 25; Grem2^−/−^, 1,201.66 ± 101.33; *n* = 24; *p* = 0.0035_r_). In addition, the total (data not shown) or percentage of time spent in the center of the open field during the 20 min initial period was significantly lower in *Grem2^−/−^* mice as well (Extended Data Fig. 4-1; WT, 42.41% ± 3.06%; *n* = 25; Grem2^−/−^, 33.20% ± 3.39%; *n* = 24; *p* = 0.0496_s_). These behaviors appear to be strictly anxiogenic, as tests for motivation, such as the novelty suppressed feeding test did not show significant differences between WT and *Grem2^−/−^* mice (Extended Data Fig. 4-1; WT, 587.90 ± 86.61 s; *n* = 11; Grem2^−/−^, 577.60 ± 92.28 s; *n* = 10; *p* = 0.9365_t_).

**Figure 4.**
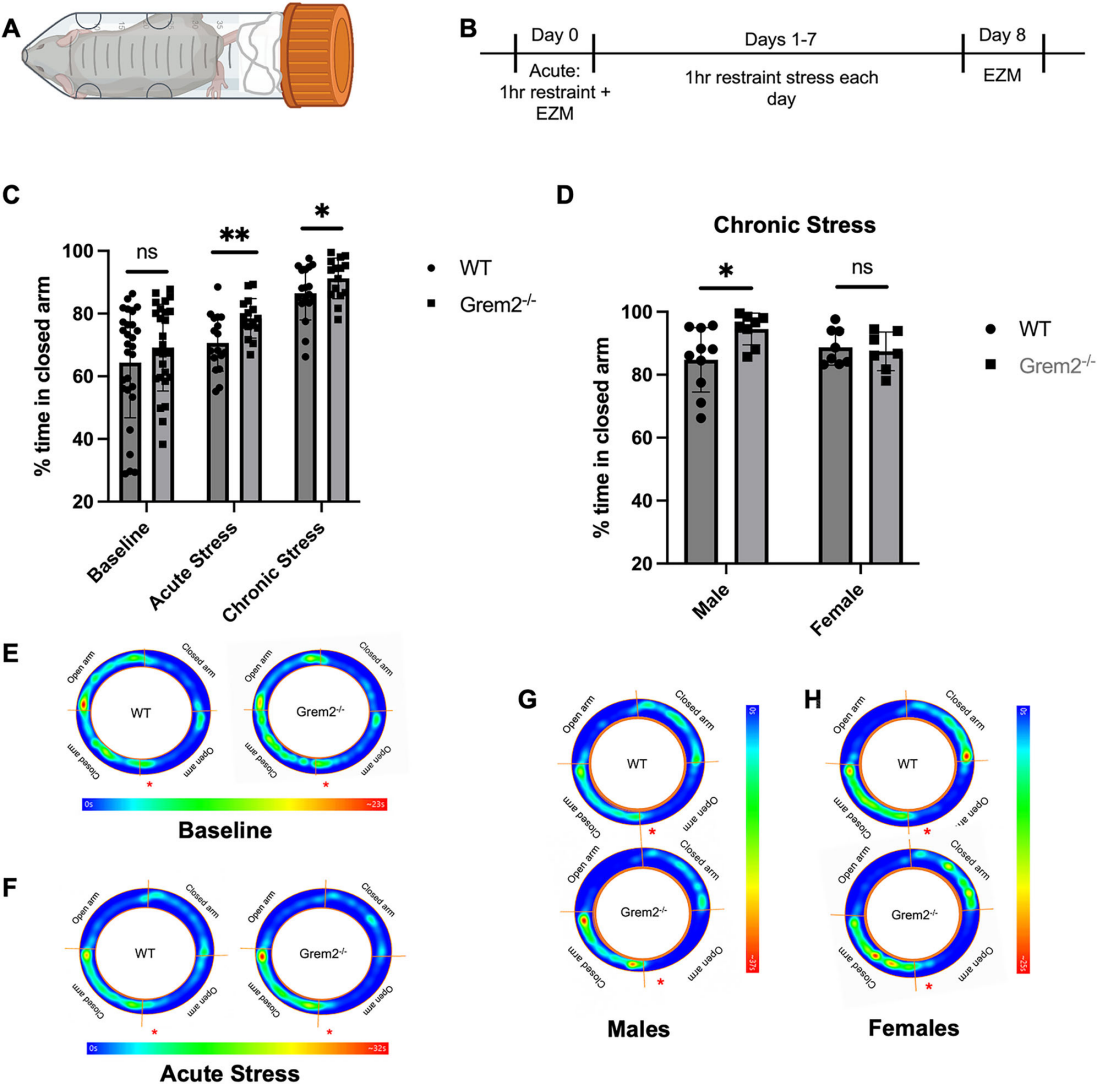
*Grem2^−/−^* mice have an anxiety phenotype that becomes more pronounced in stressed conditions. ***A***, ***B***, Mice underwent restraint stress in 50 ml conical tubes that restricted limb movement (***A***) following an 8 d timeline (***B***). ***C***, *Grem2^−/−^* mice spent more time on average in the closed arm of the EZM at the baseline, but differences were not significant. With the addition of acute and chronic stress, *Grem2^−/−^* mice spent significantly more time in the closed arms of the EZM. ***D***, There is a significant interaction between sex and genotype in the chronic stress paradigm, with male *Grem2^−/−^* mice showing the greatest anxiety phenotype in response to stress. ***E–H***, Average heat maps of WT and *Grem2^−/−^* mice on the EZM platform at the baseline (***E***), in the acute stress paradigm (***F***), males in the chronic stress paradigm (***G***), and females in the chronic stress paradigm (***H***; * represents entry point). Extended Data on baseline measurements are included in Extended Data Figure 4-1.

Due to this trend, and because of the role of BMP signaling and neurogenesis in response to stress (Gould et al., 1997; Malberg and Duman, 2003; Pham et al., 2003; Mirescu and Gould, 2006; Czéh et al., 2007; Mori et al., 2020), we placed mice under acute and chronic restraint stress to assess the ability of *Grem2^−/−^* mice to mitigate their anxiety under stressful scenarios (Fig. 4*A*,*B*). In both the acute and chronic restraint stress paradigms, *Grem2^−/−^* mice spent significantly more total (data not shown) or percentage of time in the closed arms than WT counterparts, suggesting higher levels of anxiety (Fig. 4*C*–*H*; acute, WT, 70.65% ± 2.19%; *n* = 17; Grem2^−/−^, 78.48% ± 1.63%; *n* = 15; *p* = 0.0087_u_/chronic, WT, 86.52% ± 2.01%; *n* = 17; Grem2^−/−^, 91.21% ± 1.678%; *n* = 15; *p* = 0.0302_v_). Controls were run following the same timeline as stressed mice without the addition of restraint stress to control for changes associated with memory. The differences seen between the runs on Day 0 and on Day 8 of these control mice in both total ambulation and time spent in the closed arms were not significantly different between *Grem2^−/−^* and WT mice. There results suggest that the observed behavioral differences can be mainly attributed to anxiety.

10.1523/ENEURO.0213-23.2024.f4-1 Figure 4-1 ***Grem2^-/-^* mice show evidence of heightened baseline anxiety, but levels are not significant. A,B)** Total ambulatory distance is not different between *Grem2^-/-^* and WT mice in a locomotor paradigm, though *Grem2^-/-^* mice display a significantly different activity pattern that is indicative of heightened preliminary freezing due to anxiety. **C)** Percentage of time spent in the center of the open field locomotor box is significantly higher in *Grem2^-/-^* mice for the first 20 minutes of testing. **D)** *Grem2^-/-^* mice do not show motivational deficits, with latency to first bite in NIFS testing not being significantly different from WT mice. Download Figure 4-1, TIF file.

### The role of Grem2 on mitigation of anxiety in response to chronic stress is sex-dependent

Interestingly, the anxiety effect in the chronic stress paradigm showed a significant interaction of sex when tested with a two-way ANOVA (*p* = 0.0407_w_). Males showed a more prominent effect of the lack of Grem2 on anxiety behaviors than females, with male *Grem2^−/−^* mice spending significantly more time in the closed arms than WT male mice (Fig. 4*D*,*G*,*H*; females, WT, 88.73% ± 2.01%; *n* = 17; Grem2^−/−^, 89.00% ± 1.89%; *n* = 15; *p* = 0.9624_x_; males, WT, 84.74% ± 3.23%; *n* = 17; Grem2^−/−^, 94.52% ± 1.78%; *n* = 15; *p* = 0.0193_y_).

**Figure 5.**
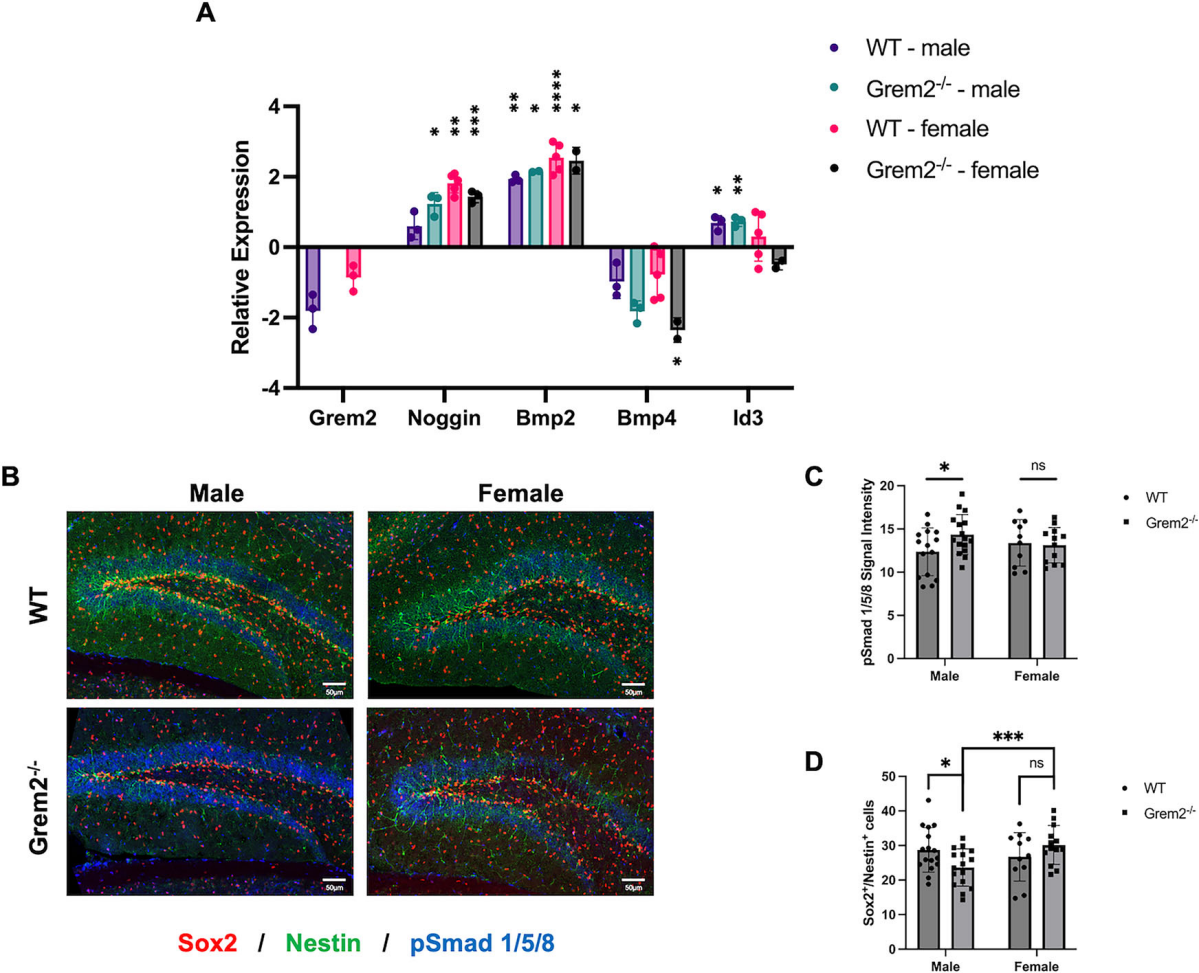
*Grem2^−/−^* male mice have increased BMP signaling in the DG following chronic stress, which is correlated with decreased neurogenesis. ***A***, qPCR analysis shows sex- and genotype-specific changes in RNA expression of markers of BMP signaling components following stress. Results are presented as logarithmic values of stress/nonstress expression level ratios. ***B***, Representative images of DG of chronically stressed *Grem2^−/−^* and WT mice of both sexes, staining for pSmad1/5/8, Nestin, and Sox2. Extended Data of single channel images of Sox2, Nestin, and pSmad1/5/8 immunofluorescence staining are included in Extended Data Figure 5-1. ***C***, ***D***, Male, but not female, *Grem2^−/−^* mice have significantly higher levels of canonical BMP signaling and fewer Sox2^+^/Nestin^+^ cells following chronic stress compared with WT controls (images taken between the bregma −2.35 and −2.48 mm).

Following the chronic stress behavioral paradigm, hippocampi of WT and *Grem2^−/−^* mice from both sexes were analyzed for levels of BMP signaling as well as NSC proliferation. Hippocampi were resected, and RNA levels of *Grem2*, *Noggin*, *Bmp2*, *Bmp4*, and *Id3* were quantified by qPCR and compared with pre-stress levels of sex- and genotype-matched controls (WT-male-NoStress/WT-male-Stress/Grem2^−/−^-male-NoStress/Grem2^−/−^-male-Stress/WT-female-NoStress/WT-female-Stress/Grem2^−/−^-female-NoStress/Grem2^−/−^-female-Stress, *n* = 4/3/6/3/5/5/5/3). We found that stress led to decreases in *Grem2* among WT animals, but values were borderline significant (*p*_male _= 0.0700_z_; *p*_female _= 0.0509_aa_). Stress caused a significant increase in Noggin among WT (*p* = 0.0043_ab_) and *Grem2^−/−^* (*p* = 0.0007_ac_) females. While Noggin was also upregulated in *Grem2^−/−^* males (*p* = 0.0200_ad_), the increase was not as striking and was not significant in WT males (*p* = 0.0960_ae_). BMP signaling was significantly altered following stress as well. *Bmp2* levels were significantly increased in all samples (*p*_WT-male _= 0.0013_af_; *p*_WT-female _< 0.0001_ag_; *p*_Grem2−/−male _= 0.0182_ah_; *p*_Grem2−/−female _= 0.0335_ai_), while *Bmp4* levels were significantly decreased only in *Grem2^−/−^* females (*p* = 0.0403_aj_). Lastly, Id3 levels, a canonical BMP signaling target, were only significantly increased in WT males (*p* = 0.0440_ak_) and more significantly in *Grem2^−/−^* males (*p* = 0.0016_al_; Fig. 5*A*). To investigate how these complex changes in the expression of BMP ligands and antagonists affect BMP signaling in the hippocampus following stress, DGs were stained with antibodies recognizing pSmad1/5/8, a marker of canonical BMP signaling, and showed a significant increased intensity in pSmad1/5/8 in only male *Grem2^−/−^* mice (*Grem2^−/−^* males, +2.00 ± 0.11 intensity; *n* = 4/*N* = 16; *p* = 0.0384_am_; Fig. 5*A*,*B*). In addition, proliferative cells in the SGZ were marked by Sox2^+^/Nestin^+^ double staining. In male *Grem2^−/−^* mice, the number of proliferative NSCs in the SGZ was significantly decreased (WT, 38.25 ± 3.83 cells; *n* = 4/*N* = 16; Grem2^−/−^, 27.125 ± 3.16 cells; *n* = 4/*N* = 16; *p* = 0.0328_an_), which correlated with increased BMP signaling (Fig. 5*A*,*C*). When comparing both sexes together, a two-way ANOVA identified a strong interaction between signal intensities and sex (*p* = 0.0026_ao_).

10.1523/ENEURO.0213-23.2024.f5-1 Figure 5-1 Single channel images of Sox2, Nestin, and pSmad1/5/8 immunofluorescence staining in the DG of WT and *Grem2^-/-^* mice of both sexes highlight the increase in pSmad1/5/8 intensity and corresponding decrease in Sox2 and Nestin that is evident in *Grem2^-/-^* males. [*Images taken between Bregma -2.35 – -2.48  mm*]. Download Figure 5-1, TIF file.

### *Grem2^−/−^* mice suffer from increased susceptibility to and higher severity of seizures

**Figure 6.**
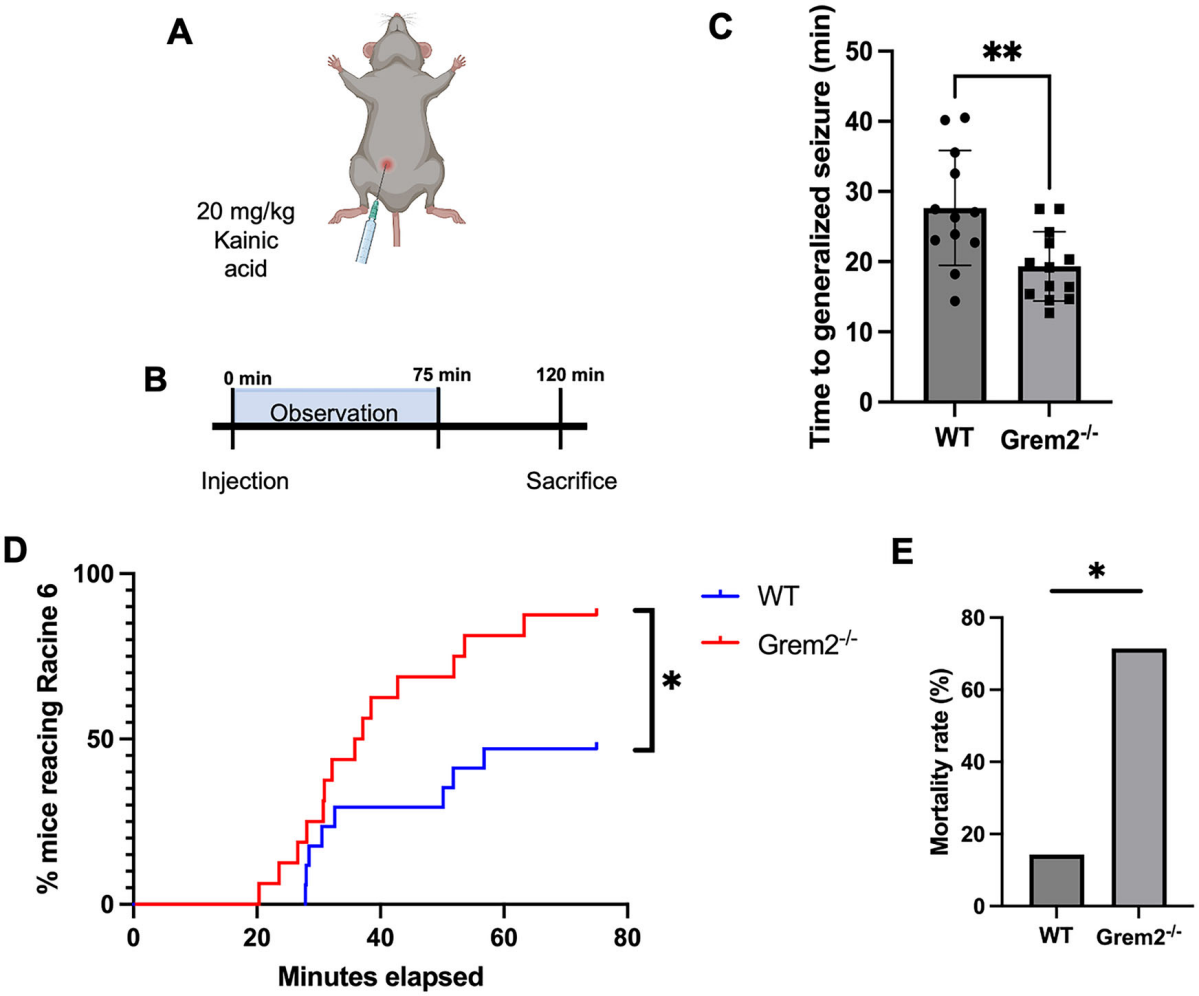
*Grem2^−/−^* mice suffer from increased susceptibility to and severity of seizures. ***A***, ***B***, Mice were injected with 20 mg/kg of KA with subsequent behavioral observation for 75 min before being killed at 120 min (***B***). ***C***, *Grem2^−/−^* mice reach generalized tonic seizure 12 min faster on average than WT counterparts. ***D***, Significantly more *Grem2^−/−^* mice reach the highest severity level of seizure (Racine 6) in 75 min. ***E***, *Grem2^−/−^* mice suffered from higher mortality at 120 min postinjection.

Aberrant hippocampal structure and neurogenesis are also hallmarks of epileptogenesis (Cho et al., 2015; Blumcke et al., 2017; J. Y. W. Liu et al., 2020). Given the observed morphological and proliferative deficits in *Grem2^−/−^* mice described above, we examined whether *Grem2^−/−^* mice were more susceptible to seizures than control WT mice. By observation alone, *Grem2^−/−^* mice did not exhibit convulsions in the absence of a chemical challenge. To test for seizure susceptibility, mice were injected with 20 mg/kg of KA, a kainate receptor agonist that leads to excitotoxicity and epileptogenesis (Fig. 6*A*,*B*). Upon observation, *Grem2^−/−^* mice reached generalized seizure on average 8.3 min faster than WT mice (Fig. 6*C*; WT, 27.66 ± 2.11 min; *n* = 15; Grem2^−/−^, 19.33 ± 1.27 s; *n* = 15; *p* = 0.0024_ap_). In addition, the progression to Racine 6 level seizures—the highest level of seizure marked by wild jumping and vocalizations (Racine, 1972; Van Erum et al., 2019)—was significantly faster and more pronounced in *Grem2^−/−^* mice, with 87.5% reaching this level compared with only 47.1% of WTs in the allotted 75 min timeframe (Fig. 6*D*; *p* = 0.0187_aq_). Furthermore, the 120 min mortality rate was significantly higher in *Grem2^−/−^* (*n* = 5/7) mice than WT (*n* = 1/7) mice, suggesting *Grem2^−/−^* mice are susceptible to higher severity of seizures upon chemical challenge (Fig. 6*E*; *p* = 0.0308_ar_).

## Discussion

Here, for the first time, we show that Grem2 has an important role in the adult brain. Specifically, our results show that mice lacking Grem2 suffer morphological and proliferative deficits in their hippocampus and behavioral abnormalities. The importance of proper regulation of BMP signaling in the brain has been previously recognized, with much of the focus on the inhibitor Noggin. Our results show that Grem2 is expressed in comparable levels with Noggin in the hippocampus and that there is no compensatory upregulation of Noggin, or other BMP antagonists, in the absence of Grem2. This suggests that Grem2 has a unique role in the brain from other BMP antagonists, which is further supported by the deregulation of BMP signaling in hippocampal samples, as well as the molecular, cellular and behavioral deficits observed in *Grem2^−/−^* mice.

Specifically, we found that mice lacking Grem2 exhibit NSC proliferative deficits in adult hippocampus, consistent with previous studies showing that overexpression of BMP signaling leads to a decrease in hippocampal NSC proliferation (Shou et al., 1999; Lim et al., 2000; Bonaguidi et al., 2008; Tang et al., 2009; Mira et al., 2010; Bond et al., 2012, 2014; Brooker et al., 2017; Díaz-Moreno et al., 2018). Histological analyses of brain sections from WT and *Grem2^−/−^* mice revealed that the total number of NSCs in the DG of adult *Grem2^−/−^* mice is decreased, suggesting that the pool of NSCs is depleted in the absence of Grem2. Furthermore, the percentage of Sox2^+^ cells that are actively dividing is also decreased, indicating that NSCs in the adult hippocampus are more quiescent in *Grem2^−/−^* mice. These results are consistent with previous studies which suggested that inhibition of BMP signaling increases neurogenesis, while administration of BMP ligands increases quiescence of NSCs (Mira et al., 2010; Bond et al., 2012, 2014; Le Dréau, 2022).

We have also documented a decrease in Dcx^+^ cells in the *Grem2^−/−^* DG. This near 50% reduction in immature neuroblasts correlates with the decrease in proliferative cells, suggesting that the proliferative deficit accounts, at least in part, for the decreased numbers of immature cells rather than solely deficits in cell maturation.

Decrease in NSC proliferation was stronger in the ventral DG, suggesting a region-specific effect of Grem2. Deficits in ventral hippocampal neurogenesis have been implicated in many anxiety phenotypes (David et al., 2009; Schloesser et al., 2014; Yun et al., 2016). We show that in correspondence with evident NSC proliferative deficits, stress-induced anxiety is significantly increased in the absence of Grem2. Stress plays an important role in the hippocampus, with stress causing a marked decrease in hippocampal neurogenesis (Gould et al., 1997; Czéh et al., 2001, 2002, 2007; Tanapat et al., 2001; Fowler et al., 2002; Malberg and Duman, 2003; Pham et al., 2003; Simon et al., 2005; Zeng et al., 2007; F. Murray et al., 2008; Jorgensen et al., 2019). It is hypothesized that this neurogenic decrease is a direct result of stress-induced BMP4 overexpression in the hippocampus (Mori et al., 2020). Administration of anxiolytics causes an increase in hippocampal neurogenesis, and it has been shown that the absence of neurogenesis or BMP inhibition of adult-born neurons in the DG blocks the anxiolytic effects of these medications (Malberg et al., 2000; Santarelli et al., 2003; Surget et al., 2008, 2011; Boldrini et al., 2009; Perera et al., 2011; Tunc-Ozcan et al., 2022). Adult-born neurons inhibit mature granule cells via activation of GABAergic interneurons in the DG, suggesting hippocampal neurogenesis acts to confer resilience to chronic stress (Ikrar et al., 2013; Drew et al., 2016). Therefore, it is likely that the increase in BMP signaling and decrease in hippocampal NSC proliferation seen in *Grem2^−/−^* mice contributes to heightened anxiety phenotypes. Furthermore, we observed a stronger manifestation of neurogenic deficits in the ventral hippocampus, which is more commonly thought to mediate anxiety-related behaviors (Fanselow and Dong, 2010; Strange et al., 2014; Jimenez et al., 2018).

We documented a sex-specific role of Grem2 in anxiety under chronic stress conditions. It appears that males are more reliant on Grem2 in mitigating anxiety in response to stress. We showed that males lacking Grem2 experienced significantly heightened canonical BMP signaling compared with both male and female WT controls and female *Grem2^−/−^* mice, which correlated with decreased neurogenesis in the SGZ. This finding corresponds with evidence of sex differences in the expression of BMP ligands and antagonists in the brain (Omrčen et al., 2021). It was shown that in stressed conditions, there is a significant correlation between Gremlin expression and cell proliferation in the DG in males, while females show a stronger correlation with Noggin (Omrčen et al., 2021). Interestingly, our data show that stress led to a dramatic induction of Noggin expression in females and that this induction was attenuated in males, supporting the notion that inhibition of BMP signaling in males is more reliant on members of the Gremlin family than in females, which may explain the increased anxiety observed in *Grem2^−/−^* males under stress conditions.

We also discovered that *Grem2^−/−^* mice suffer from greater susceptibility and severity of seizures upon administration of a chemical proconvulsant. The observed morphological scattering in the DG hilus of CA3 pyramidal networks in *Grem2^−/−^* mice may contribute to the increased susceptibility and severity of seizures seen following KA challenge. Granule cell dispersion of adult-born granule cells following acute status epilepticus (SE) contributes to epileptogenesis by aberrantly integrating into the CA3 network and synchronizing with CA3 epileptiform bursts (Scharfman et al., 2000). It is thus possible that aberrant CA3 migration toward the hilar-granule border may form potential recurrent networks with DG granule cells. This could account for the decreased latency to seizure following chemical challenge, as well as the increased severity of epileptic seizures in *Grem2^−/−^* mice. Future studies examining electrophysiological properties of DG granule cells for evidence of epileptiform burst activity, as well as staining of CA3 collaterals, would be necessary to confirm this possibility. While no evidence of seizure activity or mossy fiber sprouting (data not shown) was seen in nonchallenged animals, it is possible that following nonfatal SE as administered by KA *Grem2^−/−^* mice may be more susceptible to mossy fiber sprouting and recurrent epileptic activity.

Future research is also needed to determine the integration of the Grem2-regulated network with molecular mechanisms of hippocampal neurogenesis and overall structure. New agents targeting distinct BMP signaling components are being developed and may be tested for their effects on specific *Grem2^−/−^* phenotypes in the brain. Lastly, detailed developmental studies could determine whether the hippocampal deficits exclusively affect the adult brain or are due to abnormal developmental processes.

Taken together, our results have identified Grem2 as an important new regulator of the BMP signaling network in the hippocampus, especially in adult hippocampal neurogenesis. BMP levels in the hippocampus are increased following stress, and hippocampal BMP signaling is an important target for antidepressant and antianxiety medications (Brooker et al., 2017; Mori et al., 2020; Tunc-Ozcan et al., 2022). Increased BMP signaling in the hippocampus is also associated with epilepsy and seizure development, through modulation of neurogenesis (Parent et al., 1997, 2006; Ribak et al., 2000; Scharfman et al., 2000, 2003; Walter et al., 2007; Yin et al., 2007; Koyama et al., 2012; Pun et al., 2012; Cho et al., 2015). Therefore, our findings provide novel mechanistic insights in hippocampal structure and function, as well as a putative novel strategic target for therapeutic interventions in anxiety and epilepsy.

## Synthesis

Reviewing Editor: Viji Santhakumar, University of California Riverside Decisions are customarily a result of the Reviewing Editor and the peer reviewers coming together and discussing their recommendations until a consensus is reached. When revisions are invited, a fact-based synthesis statement explaining their decision and outlining what is needed to prepare a revision will be listed below. The following reviewer(s) agreed to reveal their identity: Chian-Yu Peng.

Synthesis: This study support an anxiolytic effect of BMP signaling inhibition, and advances the field in providing an additional molecular mechanism by which BMP signaling is regulated to maintain affective behaviors. The manuscript offers unique and exciting insight into the difference between the stress response in males and females. The manuscript has been strengthened by the revisions. Few additional revisions are recommended.

Specific Edits

1) New qPCR data bar graph in figure 5a demonstrates relative change to pre-stress levels of the same experimental group. Although the included statistical table contains details of the statistical methods and exact p values, it is recommended that error bars (ideally with individual data points) be included for each sample in bar graph to more clearly illustrate the data distribution.

2) While NeuN quantification addresses in the hilus/CA3a region suggest disorganization of the CA3 pyramidal neurons, it does not exclude the possibility that other NeuN+ neurons in the hilus, including interneurons and mossy cells, contribute to the observed phenotype. This limitation needs to be discussed.

3) The inclusion of the ventral DG analysis in Figure 3-1 is a nice addition that indicates a differential role for Grem2 in the division of ventral DG NPCs. However, the revised results section emphasized the lack of difference between dorsal and ventral DG of the same genetic background. The significant difference between pHH3+/Sox2+ in the ventral DG of WT and Grem2 should be mentioned in the results section, and the implications should be discussed.

4) Please adjust figure 2 and sf5 to present the WT data before the Grem2 data. This will make the presentation of data more consistent and easier to follow.

5) Please consider simplifying title to avoid listing all outcomes.
