# Supplementary figures and images for: BMP Antagonist Gremlin 2 Regulates Hippocampal Neurogenesis and Is Associated with Seizure Susceptibility and Anxiety
Source: eNeuro. 2024 Oct 16;11(10):ENEURO.0213-23.2024. doi: 10.1523/ENEURO.0213-23.2024 (PMC11493175; doi:10.1523/ENEURO.0213-23.2024)

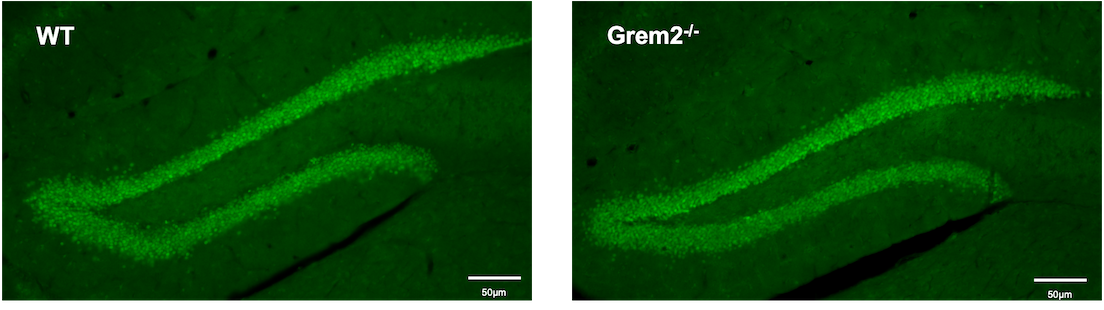

Supplement: Figure 2-1 — Prox1 staining shows no evidence of ectopically migrated hilar granule cells in WT or Grem2-/- mice. [Images taken at Bregma -1.755 mm]. Download Figure 2-1, TIF file. [file eneuro-11-ENEURO.0213-23.2024-s002.tif]

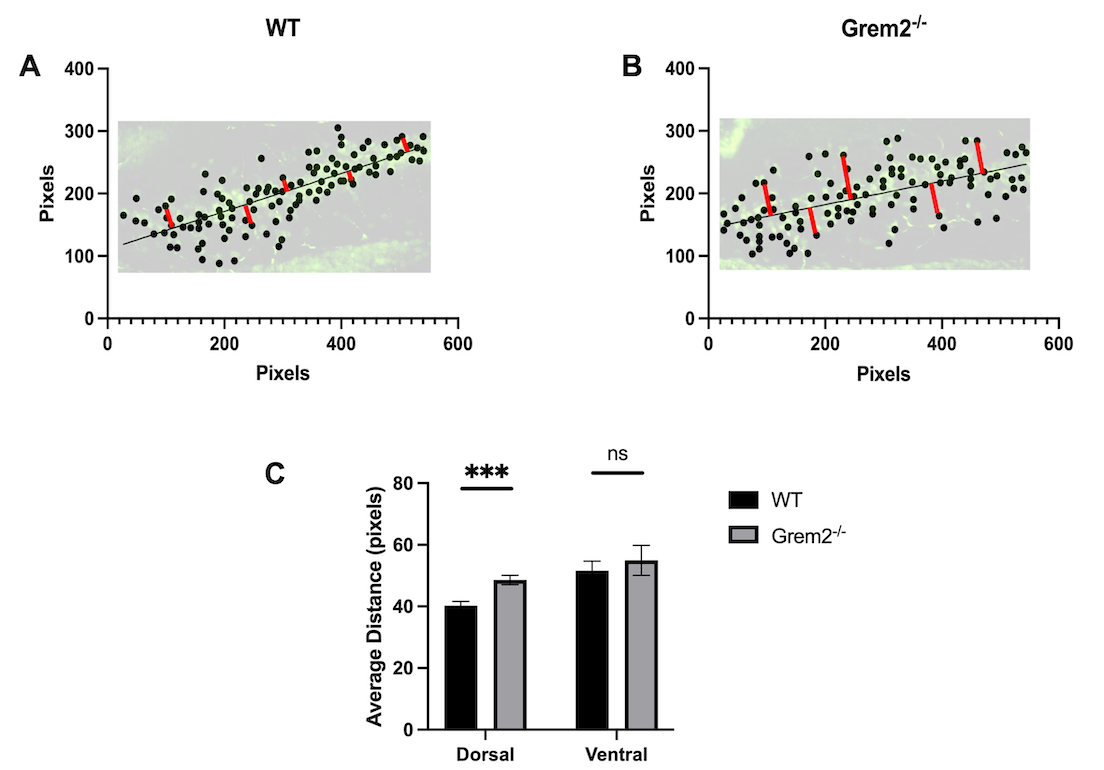

Supplement: Figure 2-2 — Scatter plots of NeuN+ cell location within the dentate hilus and analysis of scatter in dorsal and ventral hippocampi. A-B) Scatter plots of NeuN+ cell location super imposed over flipped hippocampi images from Fig2. Red line denotes example perpendicular distances calculated via equation described in the text. C) Increased scatter was seen in Grem2-/- mice in both dorsal and ventral hippocampi but was only significant in the dorsal hippocampus. Download Figure 2-2, TIF file. [file eneuro-11-ENEURO.0213-23.2024-s003.tif]

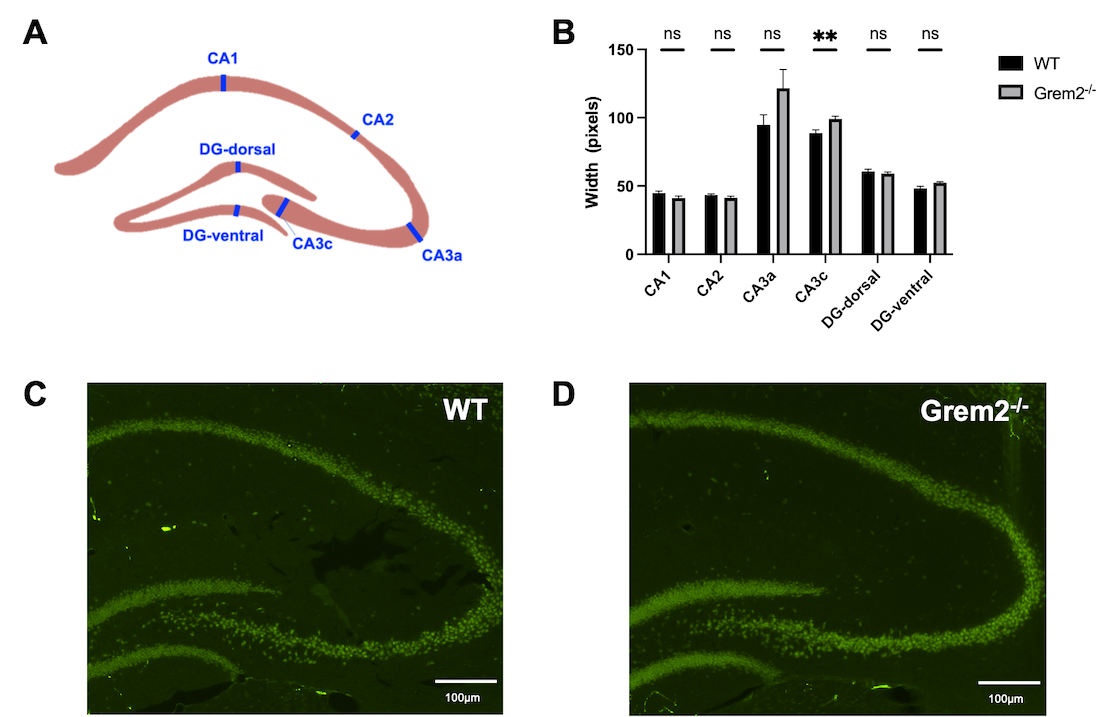

Supplement: Figure 2-3 — Grem2-/- hippocampal morphology remains intact outside of the hilar CA3 region. A) Diagram denoting where width measurements were taken along the CA and DG axes. B) There is no significant difference in width outside of the CA3c region in Grem2-/- mice when compared to WT controls. C-D) Example images of hippocampi taken at 10x magnification in WT (C) and Grem2-/- (D) mice. [Images taken at Bregma -1.755 mm]. Download Figure 2-3, TIF file. [file eneuro-11-ENEURO.0213-23.2024-s004.tif]

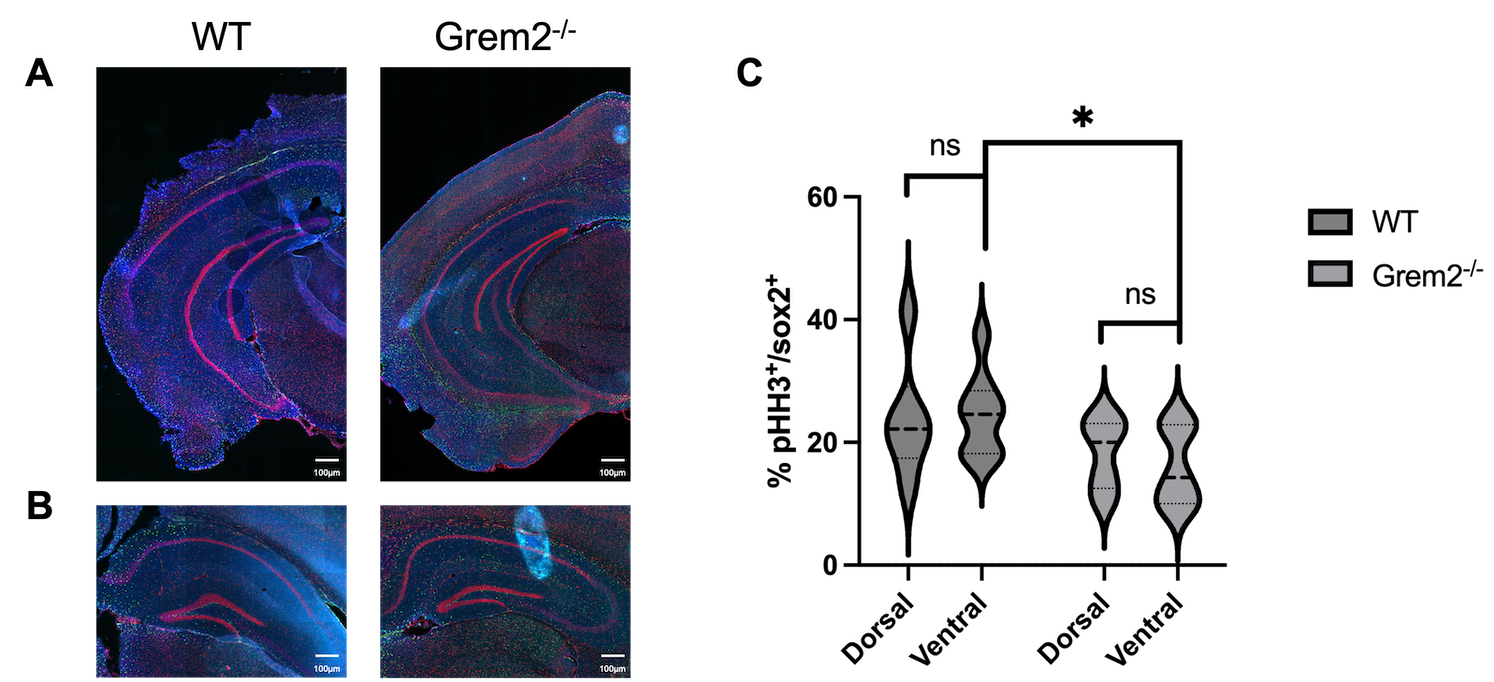

Supplement: Figure 3-1 — Proliferative differences are consistent across the dorsal-ventral axis of the DG in Grem2-/- and WT mice, but ventral proliferative deficits seems to drive overall decrease. A,B) Example images of ventral (A) and dorsal (B) hippocampi in WT and Grem2-/- mice stained for DAPI (blue), Sox2 (red), and pHH3 (green). C) There is no difference in dividing cells between the dorsal and ventral SGZ in either WT or Grem2-/- mice. However, the strongest difference appears to be when comparing proliferation within the ventral SGZs. [Ventral images taken between Bregma -3.28 – -3.48; Dorsal images taken between Bregma -2.055 mm – -2.255]. Download Figure 3-1, TIF file. [file eneuro-11-ENEURO.0213-23.2024-s005.tif]

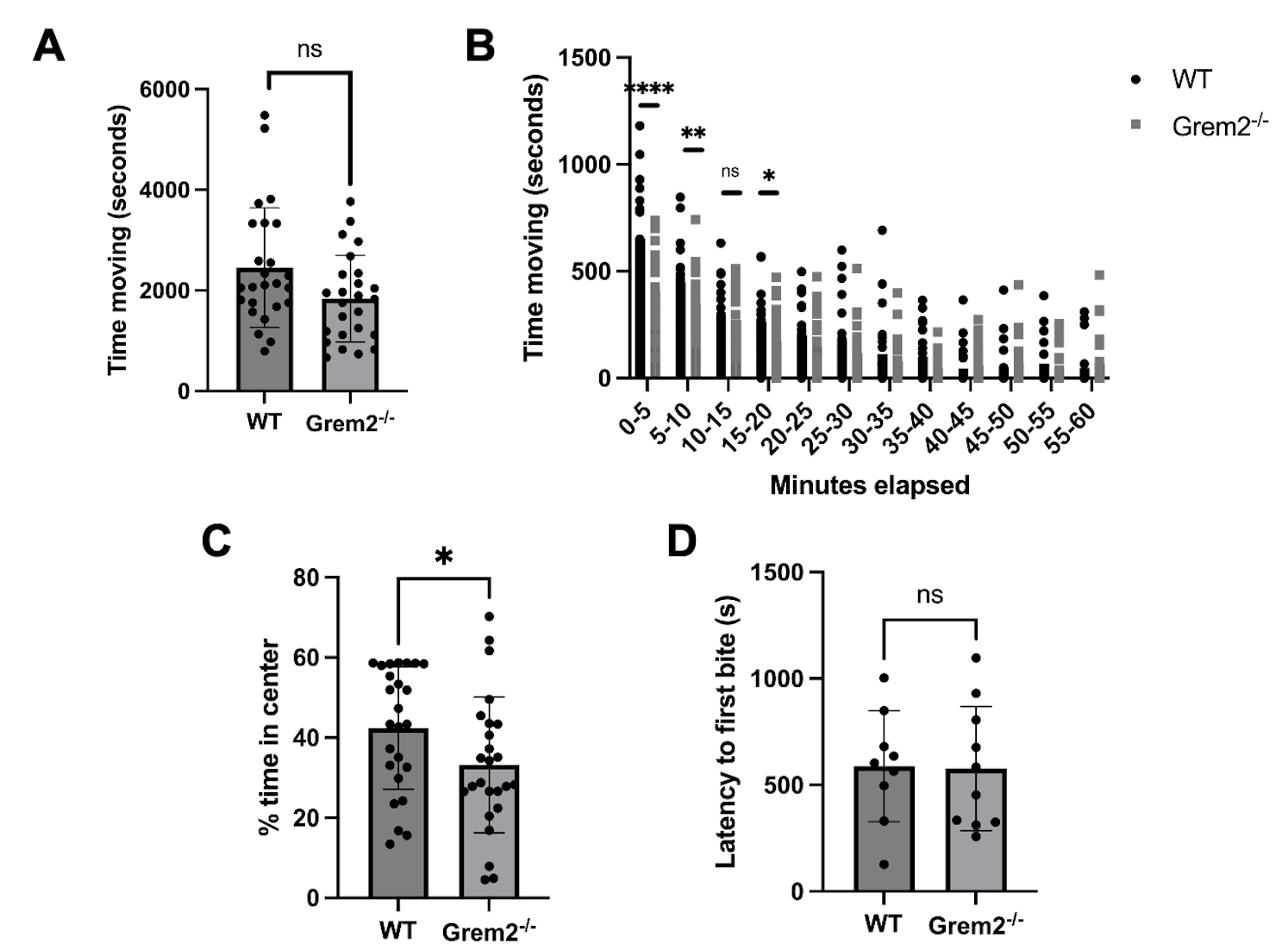

Supplement: Figure 4-1 — Grem2-/- mice show evidence of heightened baseline anxiety, but levels are not significant. A,B) Total ambulatory distance is not different between Grem2-/- and WT mice in a locomotor paradigm, though Grem2-/- mice display a significantly different activity pattern that is indicative of heightened preliminary freezing due to anxiety. C) Percentage of time spent in the center of the open field locomotor box is significantly higher in Grem2-/- mice for the first 20 minutes of testing. D) Grem2-/- mice do not show motivational deficits, with latency to first bite in NIFS testing not being significantly different from WT mice. Download Figure 4-1, TIF file. [file eneuro-11-ENEURO.0213-23.2024-s006.tif]

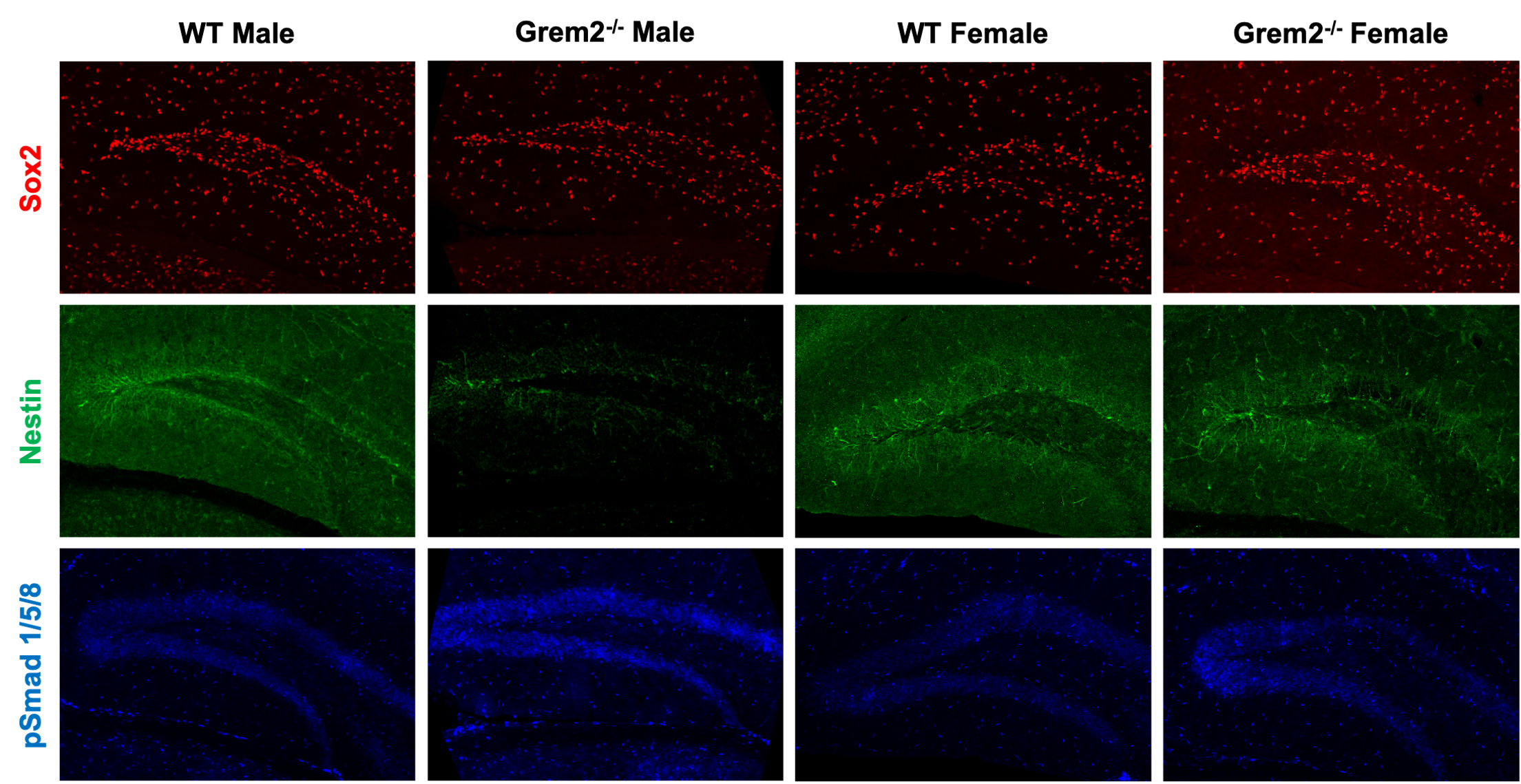

Supplement: Figure 5-1 — Single channel images of Sox2, Nestin, and pSmad1/5/8 immunofluorescence staining in the DG of WT and Grem2-/- mice of both sexes highlight the increase in pSmad1/5/8 intensity and corresponding decrease in Sox2 and Nestin that is evident in Grem2-/- males. [Images taken between Bregma -2.35 – -2.48 mm]. Download Figure 5-1, TIF file. [file eneuro-11-ENEURO.0213-23.2024-s007.tif]
